# Effect of behavioural sampling methods on local and global social network metrics: a case-study of three macaque species

**DOI:** 10.1098/rsos.231001

**Published:** 2023-12-06

**Authors:** Stefano S. K. Kaburu, Krishna N. Balasubramaniam, Pascal R. Marty, Brianne Beisner, Kevin Fuji, Eliza Bliss-Moreau, Brenda McCowan

**Affiliations:** ^1^ School of Animal Rural & Environmental Sciences, Nottingham Trent University, Southwell NG25 0QF, UK; ^2^ School of Life Sciences, Faculty of Science and Engineering, Anglia Ruskin University, Cambridge CB1 1PT, UK; ^3^ Nature and Animal Park Goldau, Goldau 6410, Switzerland; ^4^ Animal Resources Division, Emory National Primate Research Center, Emory University, 16 Atlanta, GA 30329, USA; ^5^ Department of Population Health & Reproduction, School of Veterinary Medicine, University of California, Davis CA 95616, USA; ^6^ Department of Psychology, University of California, Davis CA 95616, USA; ^7^ California National Primate Research Center, University of California, Davis CA 95616, USA

**Keywords:** aggression, all-occurrences behaviour sampling, focal animal sampling, grooming, huddling, social network analysis

## Abstract

Social network analysis (SNA) is a powerful, quantitative tool to measure animals' direct and indirect social connectedness in the context of social groups. However, the extent to which behavioural sampling methods influence SNA metrics remains unclear. To fill this gap, here we compare network indices of grooming, huddling, and aggression calculated from data collected from three macaque species through two sampling methods: focal animal sampling (FAS) and all-occurrences behaviour sampling (ABS). We found that measures of direct connectedness (degree centrality, and network density) were correlated between FAS and ABS for all social behaviours. Eigenvector and betweenness centralities were correlated for grooming and aggression networks across all species. By contrast, for huddling, we found a correlation only for betweenness centrality while eigenvector centralities were correlated only for the tolerant bonnet macaque but not so for the despotic rhesus macaque. Grooming and huddling network modularity and centralization were correlated between FAS and ABS for all but three of the eight groups. By contrast, for aggression network, we found a correlation for network centralization but not modularity between the sampling methodologies. We discuss how our findings provide researchers with new guidelines regarding choosing the appropriate sampling method to estimate social network metrics.

## 1. Introduction

Understanding the proximate and ultimate functions of social behaviour has been a central topic across many disciplines from behavioural ecology [1], to psychology [2] and neurobiology [3]. From an ultimate perspective, work conducted in the last two decades has shown that individuals who engage in more frequent and stronger social relationships live longer [4], are better at coping with social and environmental stressors [5], and produce more offspring that are more likely to survive [6]. Interestingly, accumulating evidence suggests that fitness-related benefits can be accrued not only through direct connections (i.e. how many social partners individuals have) but also through *indirect* connections (i.e. how many social partners each social partner has) [7].

In the last two decades, social network analysis (SNA) has proven to be a powerful tool in animal behavioural ecology to measure both direct and indirect connections in social animals [8,9]. SNA represents social interactions in terms of nodes (i.e. subjects involved in the interactions) and edges (i.e. connections between nodes), and provides quantitative, data-driven approaches to evaluate biologically relevant measures of animals’ connectedness both at local (i.e. individual/node) and global (i.e. group/network) levels [9]. Given these advantages, it is perhaps not surprising that SNA has been used across different contexts to study animal social relationships, including comparisons of animal social structures [1], the social diffusion of information between group members [10], the spread of infectious disease via social interactions [11,12], and in the conservation of wildlife populations [13]. Furthermore, a broad range of studies have used SNA to investigate what individual- and group-level sociodemographic and behavioural attributes, such as individuals' sex [4], dominance rank [14], personality [15] and groups’ sizes and compositions can potentially influence animals' social interactions and emergent social structure.

While it is crucial that observed networks, defined as ‘analytical representations of a combined set (or subset) of measures of the true relationships’ [8], are as similar as possible to the real networks, namely ‘the real set of interactions between animals that integrate to form community dynamics' [8], there is increasing evidence that the correspondence between observed and real networks depends on the behavioural sampling methods employed and/or on the frequency by which animals perform the behaviour of interest [16,17]. This variation may occur because observers might miss recording some real, meaningful interactions between individuals, depending on the sampling technique used and the frequency of the behaviour performed. Since network elements are inter-dependent [8,9], the absence of one or more real connections might generate an observed network that is potentially very different from a real network [8].

To date, the majority of studies examining the effect of sampling technique on variation in the structure of social networks have largely relied on simulations [16–18]. This work has suggested that a minimum number of 10–20 observations within a given network might suffice to construct a reliable network [16–18]. For instance, by generating simulated networks, Farine & Strandburgh-Peshkin [19] showed that a minimum of 20 samples is necessary in order to have an accurate estimate of the edge weight (i.e. the rate of interaction or association between two nodes) within a network. Similarly, Davis *et al*. [16] used proximity data generated by fitting high-resolution GPS collars on free-ranging baboons (*Papio anubis*) to simulate an increase in sampling effort made through two observational methods, focal animal sampling and group scanning. The authors showed that a minimum of 10 samples per individual was necessary in order for the estimated network to be similar to the complete network. In this context, it is pivotal, however, to use real biological data to test whether the reliability of network measures depends on the sampling technique used, as sometimes simulations do not accurately reflect true, biological data [e.g. 20]. Moreover, using real datasets can also better inform researchers on how to best design their methodologies to generate reliable social networks. Notwithstanding, only a few studies to date have compared different sampling techniques using actual observations, rather than simulations. McCarthy *et al*. [21], for instance, compared network measures calculated using data recorded through camera traps and focal observations among wild chimpanzees (*Pan troglodytes*). The authors found a strong correlation in network centrality indices between the two data sets, but found differences in network density and modularity. Conversely, Canteloup *et al*. [22] found a strong correlation in both grooming and play networks between data collected via *ad libitum* sampling and those recorded through focal animal sampling among vervet monkeys (*Chlorocebus pygerythrus*). More recently, Gelardi *et al*. [23] found strong similarities between social networks calculated from direct observations and through wearable proximity sensors. Collectively, these data suggest that different sampling methods yield similar network metrics, at least for local indices, while differences may emerge for global indices.

While the studies reviewed above have been crucial to understand to what extent different sampling techniques can lead to differences in social network metrics, they also lacked a comparative component as they focused either on single animal species or on a single type of behaviour. Many group-living animal taxa, however, show both intra- and inter-species differences in group cohesion and social organization, that are largely influenced by ecological factors [24–26]. Moreover, the frequency and directionality of social interactions may vary broadly across behavioural types and socio-ecological contexts. For example, groups or species may show greater ‘despotism’ in their social structures, characterized by greater frequency and unidirectionality (from dominants towards subordinates) of agonistic interactions, but lower frequencies of prosocial behaviours that are also more preferentially directed towards sub-sets of preferred prosocial partners such as close kin [27]. Conversely, groups/species that show a more egalitarian/tolerant social system may be expected to show the opposite characteristics [27]. Crucially, it remains unclear to what extent different sampling techniques can produce similar network measurements across different groups/species that display different social systems. In order to fill this gap, our study aims to compare both local and global network measures of three different social networks (aggression, grooming and huddling) collected through two different sampling techniques, focal animal sampling (FAS) and all-occurrences behaviour sampling (ABS), from three different macaque species, rhesus (*Macaca mulatta*), long-tailed (*Macaca fascicularis*) and bonnet macaques (*Macaca radiata*).

FAS and ABS are two observational methods that are most commonly used to collect behavioural data to construct animal social networks [28,29]. FAS allows an observer to focus their attention on a specific focal subject, thus offering the opportunity to record detailed information on a wide range of behaviours, both frequent and infrequent, performed by the animal [28]. However, given that, via FAS, an observer focuses only on a single animal subject, an extended period of time is likely to be needed in order to have a big enough sample size to reliably reconstruct the social network of the whole group. Conversely, by observing the whole group, ABS may reduce the number of behaviours the observer can realistically collect, but it offers the advantage of recording interactions involving multiple individuals [28]. Such cost-benefit trade-off between these two sampling techniques is likely to be one of the main criteria behind researchers' decisions on which data collection method to use. It would, therefore, be pivotal to examine whether data collected via both methods yield similar network measurements.

Macaques are a well-suited study model to compare social network indices between different sampling techniques. The genus *Macaca* includes 22 species, that show similar social organizations with female philopatry and male dispersal, but marked inter- and intra-specific variation in their social systems [27]. For instance, while some species, such as bonnet macaques, may be typically characterized by relatively more tolerant social relationships, other species such as rhesus macaques may display relatively more despotic social systems [27]. Several other species may fall somewhere in between, with some of them, such as long-tailed macaques, classified closer to the ‘despotic’ end of this spectrum [27]. Such a broad variation of social systems makes macaques well-suited models for our aims pertaining to adopting a comparative approach to assess methodological effects of observational techniques on social networks.

Here we constructed social networks for multiple, free-living groups of macaques representing three species that are typically characterized by different social systems. Using data collected via FAS and ABS, we calculated six commonly used network measures: three local metrics (degree, eigenvector and betweenness) and three global metrics (density, modularity and centralization) [9]. We compared network indices constructed from the two types of data to each other, predicting that if network measures were robust to the type of observation technique regardless of the type of social behaviour considered or the study species, then both local and global network measures from FAS data should correlate with those indices generated using ABS data. Conversely, if the accuracy of SNA metrics is contingent on species-typical social systems, we expect: (a) network measures of affiliative behaviours (grooming and huddling) to be more strongly correlated between observation methods among bonnet macaques than among long-tailed and rhesus macaques; and (b) network measures of aggressive interactions to be more strongly correlated across observation methods among the despotic rhesus and long-tailed macaques than among the more tolerant bonnet macaques. Finally, if observers are likely to record different dyadic interactions with FAS and ABS methods, then we would expect a lack of correlation between the social metrics calculated from FAS data and those calculated from ABS data.

## 2. Material and methods

### 2.1. Study sites and subjects

The study was conducted on a total of eight social groups of macaques. Rhesus macaques were studied in the city of Shimla, in Northern India (31° 05′ N–077° 10′ E) between August 2016 and February 2018. Here, we observed a total of 92 rhesus macaques (29 males and 63 females) from three macaque groups in two different locations: one group was observed in Mall Road (hereafter ‘MG’), and two groups (‘HG’ and ‘RG’) were observed at Jakhoo temple (for more details on the study site see [30,31]). Although there were some changes in the number of adult males and females across the three groups during the study period, the majority of the individuals remained in the group for most of the study (i.e. 75% of MG macaques, 79% of RG macaques and 69% of HG macaques remained in the group for at least 1 year of data collection; electronic supplementary material, figure S1).

Long-tailed macaques were studied in Kuala Lumpur (Malaysia) between September 2016 and February 2018 (3°17′ N-101°37′ E). Here we observed a total of 79 individuals (24 males and 55 females) from three macaque groups in two locations: one group (Pirate) was observed at Batu Caves, and two groups (Entrance and Hulk) were observed at Templer Park (for more details of the study site see [32]). Although these groups were subject to some demographic changes, the majority of the individuals remained in the group throughout the study period (Pirate: 80%; Entrance: 71%; Hulk: 84%; electronic supplementary material, figure S2).

Bonnet macaques were observed in Thenmala, within the state of Kerala, in Southern India between July 2017 and May 2018 (8.9° N- 77.0° E). Here the groups were studied in two locations: one (LG) was studied at the Thenmala dam while one group (SG) was studied at the Ecotourism Recreational Area (for more details of the study site and group composition see [33]). Overall, we observed a total of 79 bonnet macaques (39 males and 40 females) and, for both groups, composition was subject to very minimal demographic changes, as the majority of the macaques remained in the group throughout the study period (LG: 71%; SG: 83%; electronic supplementary material, figure S3).

### 2.2. Data collection

Across the three study sites, and with the help of 4–5 field assistants per site, we recorded information on social grooming, huddling and aggression using both FAS and ABS. We defined grooming as the manipulation of the skin or hair of a conspecifics with the hands in order to remove debris or ectoparasites, and huddling as the ventral-ventral or ventral-dorsal physical contact between individuals, while we classified as aggression any instance of chasing, aggressive grabbing, biting, slapping or threatening. Data from the field assistants were allowed to contribute to the final data set only after they reached a Cohen reliability index ≥ 0.85.

Through FAS, we followed each adult macaque for 10 min recording any social interaction (i.e. grooming, huddling and aggression) the focal subject was involved in as well as the identity of the conspecific interaction partners of the focal animal. The order by which focal subjects were selected was randomized every day, with the aim of collecting at least two focal sessions per subject per week. ABS was conducted 12 times per week, half of them in the morning and half in the afternoon. Each ABS session lasted for 10 min. At the beginning of an ABS session, the observer would record the individuals who were visible at the time. Subsequently, throughout the session, the observer would scan the group from left to right (and vice versa) to record any new instance of social interaction and the identity of the individuals involved. At the end of this 10 min session, the observer would, again, record the individuals who were present in the group, before searching for a new sub-group and start a new 10 min session. We conducted FASs and ABSs at different times of the day so as to avoid recording the same interactions using both methods. Overall, we collected a similar amount of data for both sampling methods (electronic supplementary material, figures S4 and S5): for rhesus, we recorded an average of 143.2 and a median of 138 FAS sessions per month (RG: mean = 128.2, median = 139; HG: mean = 118.2, median = 121.5; MG: mean = 169.2, median = 174), and macaques were sampled via ABS an average of 166. 2 and a median of 165 times per month (HG: mean = 101.2, median = 83; RG: mean = 201.8, median = 166; MG: mean = 194.1, median = 193.5). Similarly, for long-tailed macaques, we recorded an average of 91.8 and a median of 97 FAS sessions per month (Pirate: mean = 88.7, median = 74; Entrance: mean = 122.2, median = 121; Hulk: mean = 66.4, median = 65.5), whereas individuals were sampled an average of 88.5 and a median of 66 times per month through ABS (Pirate: mean = 77.2, median = 79; Entrance: mean = 120.6, median = 95; Hulk: mean = 71.4, median = 55). Finally, for bonnet macaques, we recorded an average of 219.6 and a median of 207.5 FAS sessions per month (SG: mean = 154.7, median = 159; LG: mean = 284.5, median = 320), while macaques were sampled an average of 232.7 and a median of 240 times per month via ABS (SG: mean = 183.2, median = 151; LG: mean = 282.3, median =293).

### 2.3. Social network analysis

We used the data on social interactions recorded via both FAS and ABS to construct social networks. Since long-tailed macaques were observed huddling only rarely (electronic supplementary material, table S1), we excluded huddling interactions for this species from the analysis. In order to take into account the fact that individuals might have been present in the group for different lengths of time, due to new individuals joining the group or some individuals disappearing from the group, we calculated interaction frequencies by dividing the number of dyadic social interactions by either the amount of time (for FAS) or the number of sessions (for ABS) in which both members of the dyad were present in the group. We then used the *sna* and *igraph* packages in R to calculate three local and three global metrics. At local level we measured: (1) *degree centrality* which reflects the number of edges that are connected to a node and thus represents the number of direct connections each subject has [9]; (2) *eigenvector centrality*, which is the sum of centralities of a node's neighbours, thereby representing the social support or social capital of an individual through being connected to animals who are in turn well connected themselves [9,34]; and (3) *betweenness centrality*, that is the number of shortest paths that flow through a node, indicating to what extent an individual connects subgroups, or may act as a ‘hub’ for information flow through the network [9]. These network measures were rescaled in order to take into account the different group sizes, and so ranged between 0 and 1. At global level, we measured: (1) *density* which is the number of edges divided by the total possible number of edges, and so assesses to what extent animals in the network are highly connected to each other [9]; (2) *modularity*, which is measured as the difference between the observed proportion of edges that fall within subgroups and the expected value of the same quantity if edges are assigned randomly and reflects to what degree a network can be subdivided into clusters of animals that more closely interact with each other than they do with animals in other clusters [35]; and (3) *centralization*, which is the difference between the eigenvector centrality of the node with the highest eigenvector centrality of the group and the eigenvector centrality of the other group members, and represents to what extent few individuals tend to be more central within a social network [36]. While degree and density were computed as unweighted measures, without taking into account the frequency of each dyadic interaction, eigenvector, betweenness, modularity and centralization were calculated as weighted measures.

### 2.4. Data analysis

We first tested the robustness of each social network. We used two approaches to assess network robustness: we first assessed, for each data collection method and for each social behaviour, the variation in mean value of all three local network measures as well as the variation of all three global measures over time with monthly increases of data collected. We expected the curves to become progressively ‘flatter’ because, if the networks were becoming more and more stable over time, monthly variation in network measures would become smaller and smaller as observers recorded fewer and fewer new edges between nodes. Second, we followed previous approaches [19,22,37], and used bootstrapping to estimate network uncertainty, which reflects the (un)certainty with which network metrics were estimated. For each monthly data and for each social behaviour examined, the identity of the recipient was randomly reshuffled and social network metrics were re-calculated. This procedure was repeated 1000 times, eventually generating a distribution of possible values. From this distribution, we extracted the 95% confidence interval and subtracted the maximum and minimum value of this range in order to calculate the uncertainty index. We then assessed, for both sampling methods, the monthly variation of this uncertainty index, expecting this value to decline as more observations were recorded and networks would become more certain.

In order to assess whether local network measures calculated from FAS and ABS data were correlated, we ran Generalized Linear Mixed Model (GLMM) analyses with Beta error structure through the R function *glmmtmb*. In this model, ABS network measures were set as outcome variables in separate models, giving us a total of nine GLMMs. As predictors, we included FAS network measures, and species ID to account for their potential effects on network measures. We selected a Beta error structure for the GLMM models because the outcome variable could only range between 0 and 1 [38]. Finally, group identity was entered as a random factor in order to control for the non-independence of individuals from the same group. To assess whether network measures calculated using the two different methodologies were positively correlated for all species, or only for some species, we compared the Akaike Information Criterion (AIC) value of the null model (i.e. the model that included only the outcome variable and the random factor), with the model that included the predictors only as main effects, and the model that included the interaction between the FAS network measures and the species. We used the *influence_mixed* and *infIndexPlot* functions to check the presence of influential observations. The ‘performance’ package in R was used to both calculate the effect size (*R*^2^) of the GLMM model and verify that all GLMM models met the necessary assumptions of model validity (i.e. distribution of residuals, residuals plotted against fitted values). Given that network measures are not independent as an individual's network metric depends on other individuals' network positions, researchers typically use permutation to test the statistical significance of regression models [8,39]. However, recent simulations have suggested that permutation methods do not control for non-independence of the data and that GLMMs can already provide robust results [40]. Because no consensus has yet been reached on the best statistical approach when using regression models for social network data, in the main text we present the results of the GLMM analysis without permutation, while in the supplementary materials we present the results of the permutation analysis, in which we compared the estimates generated from the observed data with a distribution of estimates calculated from random networks [39]. To this end, for each best GLMM model, we conducted a post-network node-swapping randomization which generated 1000 networks from the ABS data by randomly shuffling the identity of the network nodes, and then re-ran the GLMM analysis for each of these 1000 networks. This produced a distribution of estimates from these models and we calculated one-tailed p-values by comparing the number of the random estimates that were higher than the observed estimate.

Finally, we used Pearson's correlation test to assess whether global measures calculated from FAS data significantly correlated with the measures calculated from ABS data.

R-codes and data are available in our data repository (https://figshare.com/projects/Effect_of_behavioural_sampling_methods_on_local_and_global_social_network_metrics_A_case-study_of_three_macaque_species/166205).

## 3. Results

### 3.1. Network robustness

**Figure 1.**
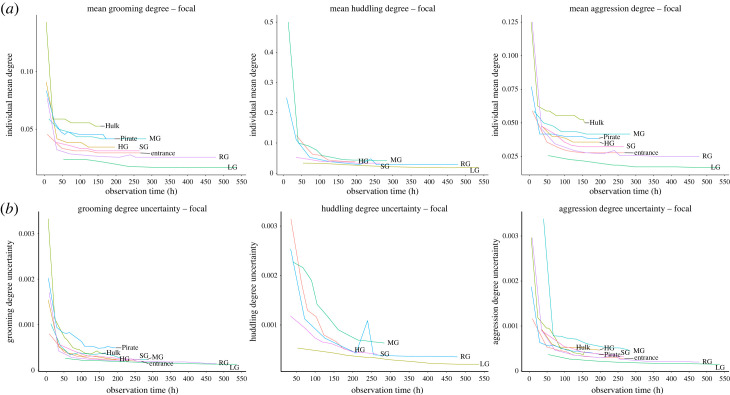
Monthly variation in individual mean grooming, huddling and aggression degree (top row) and degree uncertainty (bottom row) with progressive monthly increases in focal animal sampling observation time. Each line represents a study group. Rhesus macaque groups: RG, HG, MG; long-tailed macaque groups: Pirate, Hulk, Entrance; bonnet macaque groups: SG, LG.

Table S1 in the electronic supplementary material summarizes the total number and frequencies of social interactions recorded for all three species and for both sampling methods, while visual representations of social networks calculated from both FAS and ABS for all three behaviours examined can be found in the supplementary material (electronic supplementary material, figures S6–S13). Plotting monthly variation in network metrics (both mean local and global metrics) and their uncertainty values with monthly increases of data recorded across the three species revealed a progressive flattening of the curves for both FAS and ABS data (figure 1 and electronic supplementary material, figures S14–S24). Although network density was expected to either remain the same or increase over time, our analysis showed occasional reductions in network density values. These are likely due to small changes in demographics (e.g. if an individual disappeared from the group, the connections this individual had with other group members will have disappeared too). For both FAS and ABS, mean individual metrics flattened and uncertainty values dropped (suggesting more accuracy in the measurement) relatively early in data collection, although it required substantially more effort to achieve this when data were collected through FAS than when they were collected via ABS. More specifically, when data were collected via FAS, it took at least 50 h of observations to reach no or minimal fluctuations of local metrics and their uncertainty with progressive increase in observation time (figure 1 & electronic supplementary material, S14–S15). Conversely, when data were recorded through ABS, it took less than 10 h to reach the same result (electronic supplementary material, figures S16–S18). Furthermore, similar to the local network metrics, our analysis of global metrics and their uncertainty values shows a progressive flattening of the curves. However, we found more fluctuation over time of global metrics compared to local indices with larger fluctuations for data collected through ABS than those collected through FAS (electronic supplementary material, figures S19–S24). Furthermore, interestingly, it appears that it takes longer to reach a stability in global metrics compared to local metrics for both sampling methods. In fact, it took at least 100 h of observation time with FAS and 15 h of observation time with ABS to achieve minimal fluctuation in global metrics. Collectively, the fact that our analysis shows that variation in both local and global metrics with progressive increase in observation time reaches a plateau and that uncertainty levels decrease suggest that the social networks measures in this study are accurate and robust.

### 3.2. Grooming network analysis

**Figure 2.**
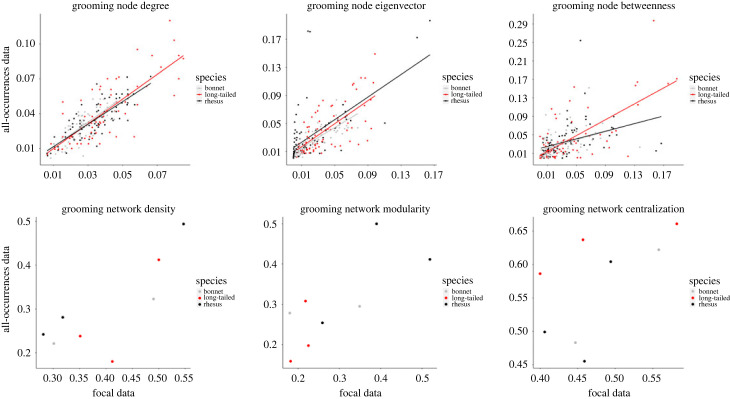
Scatterplot plotting the three local (top row) and global (bottom row) grooming network metrics calculated from all-occurrences behaviour sampling (ABS) data against those calculated from focal animal sampling (FAS) data.

The analysis of the grooming network showed a significant effect of the interaction between FAS data and species on ABS network metrics for both degree and betweenness (table 1 & electronic supplementary material, table S2). While all three species showed a positive relationship between FAS and ABS networks, this relationship was stronger for long-tailed macaques than for the other two species (figure 2). Conversely, we found a significant main effect of FAS eigenvector on ABS eigenvector (table 1 & electronic supplementary material, table S2). In other words, the macaques who were more central in the grooming network (through both direct and indirect connections) as measured by the FAS data, were also more central in the grooming network as estimated by ABS data, across all three species.

**Table 1.** Results of the GLMM analysis testing whether individuals' grooming centrality measures calculated from the focal animal sampling (FAS) data and species identity (rhesus, long-tailed, bonnet) significantly predicted grooming centrality measures calculated from the all-occurrences behaviour sampling (ABS) data. Significant predictors are indicated in bold.

predictors	estimate	s.e.	95% CI	*z*-value	*p*-value
grooming degree					
**intercept**	**−4.60**	**0.14**	**−4.88; −4.32**	**−32.07**	**< 0.001**
**FAS degree**	**38.67**	**4.55**	**29.75; 47.60**	**8.49**	**< 0.001**
**species (long-tailed versus bonnet)**	**0.35**	**0.17**	**0.02; 0.69**	**2.07**	**0.038**
species (rhesus versus bonnet)	0.19	0.18	−0.17; 0.55	1.04	0.297
species (rhesus versus long-tailed)	0.16	0.15	−0.13; 0.46	1.07	0.282
**FAS × species (long-tailed versus bonnet)**	**−14.05**	**4.89**	**−23.64 −4.47**	**−2.87**	**0.004**
FAS × species (rhesus versus bonnet)	−8.38	5.36	−18.89; 2.14	−1.56	0.118
FAS × species (rhesus versus long-tailed)	−5.68	3.36	−12.27; 0.91	−1.69	0.091
grooming eigenvector					
**intercept**	**−3.96**	**0.10**	**−4.16; −3.77**	**−40.10**	**< 0.001**
**FAS eigenvector**	**17.05**	**1.13**	**14.82; 19.27**	**15.03**	**< 0.001**
species (long-tailed versus bonnet)	0.03	0.12	−0.20; 0.26	0.25	0.800
species (rhesus versus bonnet)	−0.01	0.12	−0.24; 0.22	−0.11	0.913
species (rhesus versus long-tailed)	0.04	0.11	−0.17; 0.26	0.40	0.692
grooming betweenness					
**intercept**	**−3.70**	**0.16**	**−4.02; −3.38**	**−22.52**	**< 0.001**
**FAS betweenness**	**11.49**	**3.15**	**5.32; 17.65**	**3.65**	**< 0.001**
**species (long-tailed versus bonnet)**	**−0.56**	**0.21**	**−0.98; −0.15**	**−2.64**	**0.008**
species (rhesus versus bonnet)	0.004	0.21	−0.40; 0.41	0.02	0.985
**species (rhesus versus long-tailed)**	**−0.57**	**0.20**	**−0.96; −0.18**	**−2.86**	**0.004**
FAS × species (long-tailed versus bonnet)	5.02	3.55	−1.93; 11.98	1.42	0.157
FAS × species (rhesus versus bonnet)	−2.05	3.78	−9.47; 5.36	−0.54	0.587
**FAS × species (rhesus versus long-tailed)**	**7.07**	**2.70**	**1.84; 12.31**	**2.65**	**0.008**

For global measures, we found a significant correlation between FAS and ABS data for both grooming density (*r*_(6)_ = 0.79; *p* = 0.02) and modularity (*r*_(6)_ = 0.76; *p* = 0.03, figure 2), but not centralization (*r*_(6)_ = 0.59; *p* = 0.11, figure 2). A close look at the centralization values shows that these values were particularly different between sampling methods in one rhesus (RG) and two long-tailed macaque groups (Hulk and Entrance). In fact, when these data points were removed, we found a significant correlation between ABS and FAS centralization values (*r*_(3)_ = 0.91; *p* = 0.03).

Collectively, this analysis showed that grooming network density and modularity were both highly consistent (correlated) across sampling methods for all three macaque species, whereas we did not find evidence that grooming network centralization was correlated between ABS and FAS. This lack of correlation is likely driven by one rhesus and two long-tailed macaque groups.

### 3.3. Huddling network analysis

**Figure 3.**
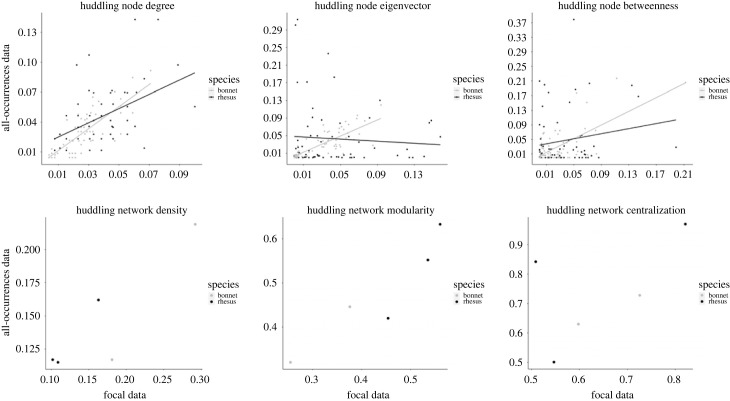
Scatterplot plotting the three local (top row) and global (bottom row) huddling network metrics calculated from all-occurrences behaviour sampling (ABS) data against those calculated from focal animal sampling (FAS) data.

The analysis of huddling network at local level showed that, for both degree and eigenvector centrality, the interaction between FAS data and species was better fit compared to the null model and the model which included only the main effects terms (table 2 & electronic supplementary material, table S5). Exploring this interaction term further revealed that, for both rhesus and bonnet macaques, FAS degree positively predicted the corresponding ABS centrality measures, but that the relationship was stronger for bonnet macaques compared to rhesus macaques (figure 3), which supports our prediction. Conversely, for huddling network eigenvector, there was a positive relationship between FAS and ABS data for bonnet, while a negative relationship for rhesus macaques (figure 3). Finally, for betweenness centrality, the model that included only the main effect was a significantly better fit compared to the model that included the interaction term (electronic supplementary material, table S5). As predicted, this model showed a positive relationship between FAS and ABS betweenness (table 2).

**Table 2.** Results of the GLMM analysis testing whether individuals' huddling centrality measures calculated from the focal animal sampling (FAS) data and species identity (rhesus and bonnet) predicted huddling centrality measures calculated from the all-occurrences behaviour sampling (ABS) data. Significant predictors are indicated in bold.

predictors	estimate	s.e.	95% CI	*z*-value	*p*
huddling degree					
**intercept**	**−4.41**	**0.17**	**−4.74; −4.06**	**−25.27**	**< 0.001**
**FAS degree**	**31.82**	**4.35**	**23.3; 40.0**	**7.31**	**< 0.001**
**species (rhesus versus bonnet)**	**0.79**	**0.25**	**0.30; 1.28**	**3.16**	**0.002**
**degree × species (rhesus versus bonnet)**	**−18.32**	**5.59**	**−29.3; −7.40**	**−3.28**	**0.001**
huddling eigenvector					
**intercept**	**−4.67**	**0.88**	**−6.40; −2.95**	**−5.31**	**< 0.001**
**FAS eigenvector**	**38.98**	**5.25**	**28.69; 49.28**	**7.42**	**< 0.001**
species (rhesus versus bonnet)	−0.66	1.12	−2.85; 1.53	−0.59	0.553
**eigenvector × species (rhesus versus bonnet)**	**−26.42**	**6.15**	**−38.48; −14.36**	**−4.30**	**< 0.001**
huddling betweenness					
**intercept**	**−3.48**	**0.26**	**−3.99; −2.96**	**−13.26**	**< 0.001**
**FAS betweenness**	**9.99**	**2.53**	**5.03; 14.95**	3.95	**< 0.001**
species (rhesus versus bonnet)	−0.38	0.25	−0.87; 0.11	−1.52	0.128

Global analysis revealed a significant correlation between ABS and FAS data for both network density (*r*_(3)_ = 0.89; *p* = 0.04) and modularity (*r*_(3)_ = 0.93; *p* = 0.02, figure 3). By contrast, we did not find a significant correlation between the two sampling methods for network centralization (*r*_(3)_ = 0.57; *p* = 0.32). Again, data from the RG group appeared to be an outlier. When this group was excluded, there was a significant correlation between ABS and FAS huddling network centralization values (*r*_(2)_ = 0.97; *p* = 0.03, figure 3).

Collectively, these results suggest that FAS and ABS yield similar, consistent network metrics for all local network metrics. At the global level, these methods yield consistent metrics for network density and modularity, while for network centralization ABS and FAS sampling methods produced similar values for all but one group.

### 3.4. Aggression network analysis

**Figure 4.**
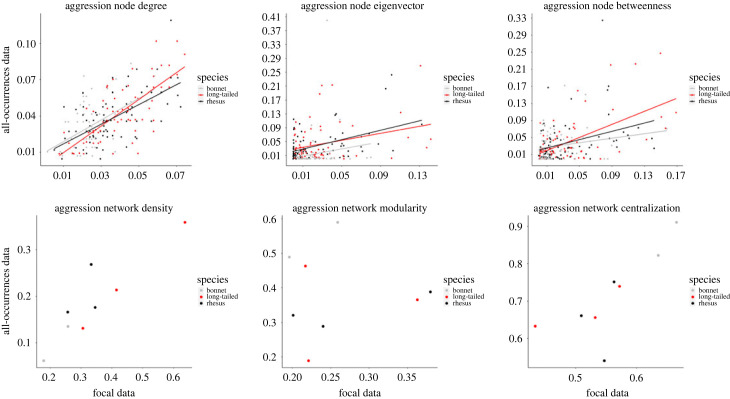
Scatterplot plotting the three local (top row) and global (bottom row) aggression network metrics calculated from all-occurrences behaviour sampling (ABS) data against those calculated from focal animal sampling (FAS) data.

The analysis of aggression network showed that, across all three local measures, the models that included the predictors as main effects only had a better fit compared to the models that included the interaction between FAS network and species (table 3 and electronic supplementary material, table S6). For all three measures, there was a positive relationship between FAS and ABS data across all three species (degree: *β* ± SE = 23.80 ± 2.02, *z* = 11.77, *p* < 0.001; eigenvector: *β* ± SE = 8.85 ± 2.18, z = 4.07, *p* < 0.001; betweenness: *β* ± SE = 11.17 ± 1.78, *z* = 6.29, *p* < 0.001; table 3; figure 4), suggesting that individuals that displayed higher aggression network degree, eigenvector and betweenness centrality values when data were collected through FAS, exhibited similar centrality values when data were collected through ABS.

**Table 3.** Results of the GLMM analysis testing whether individuals' aggression centrality measures calculated from the focal animal sampling (FAS) data and species identity (rhesus, long-tailed, bonnet) significantly predicted aggression centrality measures calculated from the all-occurrences behaviour sampling (ABS) data. Predictors that are significant are indicated in bold.

predictors	estimate	s.e.	95% CI	*z*-value	*p*
aggression degree					
**intercept**	**−4.06**	**0.09**	**−4.23; −3.88**	**−46.73**	**−46.730**
**FAS degree**	**23.80**	**2.02**	**19.84;−27.77**	**11.77**	**< 0.001**
species (long-tailed versus bonnet)	−0.06	0.09	−0.24;0.11	−0.72	0.474
species (rhesus versus bonnet)	−0.09	0.09	−0.26; 0.081	−1.02	0.309
species (long-tailed versus rhesus)	0.02	0.07	−0.12; 0.17	0.34	0.735
aggression eigenvector					
**intercept**	**−4.64**	**0.21**	**−5.05;4.22**	**−21.94**	**< 0.001**
**FAS eigenvector**	**8.85**	**2.18**	**4.58; 13.12**	**4.07**	**< 0.001**
**species (long-tailed vs bonnet)**	**1.15**	**0.23**	**0.87; 1.77**	**4.96**	**< 0.001**
**species (rhesus versus bonnet)**	**1.32**	**0.23**	**0.70; 1.61**	**5.75**	**< 0.001**
species (rhesus versus long-tailed)	−0.16	0.20	−0.56; 0.23	−0.82	0.41
aggression betweenness					
**intercept**	**−4.07**	**0.21**	**−4.48; −3.66**	**−19.56**	**< 0.001**
**FAS betweenness**	**11.17**	**1.78**	**7.69; 14.65**	**6.29**	**< 0.001**
species (long-tailed versus bonnet)	0.36	0.22	−0.06; 0.79	1.67	0.095
**species (rhesus versus bonnet)**	**0.47**	**0.21**	**0.06; 0.09**	**2.27**	**0.023**
species (rhesus versus long-tailed)	−0.10	0.20	−0.49; 0.28	−0.53	0.596

At global level, we found a significant correlation between FAS and ABS data for both aggression network density (*r*_(6)_ = 0.90; *p* = 0.002) and centralization (r_(6)_ = 0.78; *p* = 0.02; figure 4). By contrast, we found no evidence that aggression network modularity was significantly correlated between the two sampling methods (*r*_(6)_ = 0.02; *p* = 0.95).

Collectively, our results showed that, for aggressive interactions, FAS data produce similar network measures as those produced by ABS data for all local network indices (i.e. degree, eigenvector and betweenness) and for two of the three global metrics examined (i.e. density and centralization), while aggression modularity was not correlated between the two sampling methods.

Tables 4 and 5 provide a summary of the results.

**Table 4.** Summary of the results of the analysis testing the correlation of local network measures between data collected through focal animal sampling (FAS) and all-occurrences behaviour sampling (ABS).

social behaviour	social network index	significant correlation between FAS and ABS data	main effect/interaction with species
grooming	degree	yes	interaction
	eigenvector	yes	main
	betweenness	yes	interaction
huddling	degree	yes	interaction
	eigenvector	yes	interaction
	betweenness	yes	main
aggression	degree	yes	main
	eigenvector	yes	main
	betweenness	yes	main

**Table 5.** Summary of the results of the analysis testing the correlation of global network measures between data collected through focal animal sampling (FAS) and all-occurrences behaviour sampling (ABS). Rhesus macaque groups: RG, HG, MG; long-tailed macaque groups: Pirate, Hulk, Entrance; bonnet macaque groups: SG, LG.

social behaviour	social network index	significant correlation between FAS and ABS data	notes
grooming	density	yes	—
	modularity	yes	—
	centralization	no	significant correlation after excluding RG, Hulk & Entrance
huddling	density	yes	—
	modularity	yes	—
	centralization	no	significant correlation after excluding RG
aggression	density	yes	—
	modularity	no	—
	centralization	yes	—

## 4. Discussion

The overarching goal of our study was to investigate whether two commonly used data collection methods, FAS and ABS, produce similar social network measures. To this end, we compared three local (degree, eigenvector and betweenness) and three global (density, modularity and centralization) network indices for three social behaviours (aggression, grooming and huddling) in three macaque species (rhesus, long-tailed and bonnet) that display different levels of species-typical social structures.

Previous simulation-based work suggested that researchers would need to collect at least 15–20 interactions per dyad in order to construct a reliable social network [16–18]. For large groups containing many individuals and potential interactions, this would mean having to collect thousands of observations [16]. By contrast, our analysis examining variation in local and global metrics over time revealed that it took no more than a total of 50 h for data collected through FAS, and 10 h for data collected through ABS, to reach a stable network with minimal or no fluctuation of local network metric values with progressive increases in observation time. This was true across all group sizes, from the small rhesus macaque MG group, with 24 adults, to the large bonnet macaque group LG, with 60 individuals. This discrepancy is likely due to the fact that, while previous research was largely based on simulations [17,18], our study relied on actual behavioural observations. One possible reason why it takes less effort than expected to construct and estimate reliable social network measures could be that, in the attempt to establish or maintain long-term social relationships within their groups such as social bonds [6] or dominance ranks [41], animals direct social behaviours, such as grooming, huddling and aggression, towards specific group members. This means that with only a few hours of observations, individuals' network position would become apparent. Crucially, this means that species characterized by sparser and less kin-directed social interactions might require a greater sampling effort to generate a reliable social network [18]. Interestingly, it takes more observation hours (at least 100) to reach a stability in global compared to local metrics, probably because global network metrics are more sensitive to missing edges compared to local network metrics [16] and so a larger number of observations are needed to record all or most dyadic interactions, including the more infrequent ones.

Our comparison of the network metrics calculated from the two sampling methods revealed that, for grooming and aggression networks, all three local network centrality measures were significantly, positively correlated across the two behavioural sampling methods, and for all three macaque species. This suggested that methodological differences in behavioural data collection did not seem to impact node degree, eigenvector and betweenness centrality measures, regardless of species-typical social structure or social styles. By contrast, for huddling networks, only degree and betweenness centralities were correlated between the two sampling methods for both bonnet and rhesus macaques, while eigenvector centrality measures were correlated between the two sampling methods only for the tolerant bonnet macaques but not for the despotic rhesus macaques.

The analysis and comparisons of global metrics revealed that correlations between metrics calculated using the two sampling methods depended both on the species, the type of behaviour and network metric examined. In particular, for grooming behaviour, we found a positive correlation for grooming network density and modularity while grooming network centralization was correlated between FAS and ABS data only if three groups (one rhesus and two long-tailed macaque groups) were excluded from the analysis. Similarly, we found that FAS huddling network metrics correlated with the respective ABS global network metrics for density and modularity but not for centralization. Yet, when one rhesus macaque group was excluded from the analysis, we did find a correlation in huddling centralization between the two sampling methods. Finally, for aggression networks, we found a positive correlation between the two sampling methods only for network density and centralization but not for network modularity.

Collectively, our study shows that, for all social behaviours examined and for all the macaque species investigated, network attributes that measure *direct* interactions, namely degree (at local level) and density (at global level) were strongly correlated between the two sampling techniques. This indicates that researchers who are interested in assessing how many direct interactions each animal has and/or how many edges are present in the group, can employ either sampling technique regardless of the social behaviour examined or the degree of specie-specific sociality. However, despite the fact that ABS and FAS data produce comparable social network measures of direct interactions, the usefulness of SNA lies in its ability to provide measurements of animals’ *indirect* connections [7,8]. In this regard, our study showed that the correspondence between FAS and ABS network metrics largely depends on the social behaviour examined, and group- or species-typical characteristics such as social organization and emergent social structure or social style. More specifically, we found that for those social behaviours performed at high frequency, namely social grooming and aggression for all three species, and huddling for bonnet macaques, there was a strong positive relationship in eigenvector and betweenness centrality values calculated from both sampling methods. This suggests that both sampling methods yield similar local network metrics that reflect indirect connections regardless of group- or species-typical social style. In this context, ABS seems to be the most cost-effective sampling method as it requires less effort to collect more dyadic interactions.

While our findings indicate that either sampling method can be used to construct reliable social networks from frequently occurring social behaviours, they also suggest that network measures calculated from *infrequent behaviours* are especially vulnerable to the type of sampling method used. In fact, for huddling interactions, we found that eigenvector centrality was correlated between the two sampling methods only for the tolerant bonnet macaque, but not so for the despotic rhesus macaques which were observed huddling at much lower frequencies. When or where feasible, we therefore suggest the use of ABS rather than FAS in order to construct reliable social networks from infrequent behaviours as ABS allows researchers to record more dyadic interactions compared to FAS. In fact, via ABS, we collected a frequency of huddling behaviour from rhesus macaques that was nearly 5 times higher compared to the frequency of interactions recorded through FAS (see electronic supplementary material, table S1).

For prosocial behaviours (i.e. grooming and huddling), we found that FAS network centralization correlated with ABS network centralization only if one rhesus macaque (RG) and two long-tailed macaque (Hulk and Entrance) groups were excluded from the analysis. Network centralization reflects the proportion of social interactions that involve one or a few individuals, and, in macaques, variation in this index has been found to be associated with dominance rank and species' degree of tolerance/despotism [36]. In other words, in despotic species such as rhesus macaques, which exhibit marked rank relationships, social grooming tends to be largely directed towards high-ranking individuals, and so these species tend to have a highly centralized network, while in more tolerant macaque species, grooming interactions tend to be more equally distributed across dyads exhibiting, therefore, a less centralized network [36]. Here we suggest that the variation in key demographic components and the degree of social (in)stability of the study groups might explain why, for some macaque groups, network centralizations calculated from both FAS and ABS data were not correlated. In RG, for instance, some high-ranking individuals, including the dominant female, disappeared from the group during our study period. Similarly, the long-tailed macaque groups experienced several turnovers in the male dominance hierarchy. These demographic changes might have shifted the rank relationships within the study groups influencing the effect of rank on the direction of grooming interactions, affecting, thereby, grooming network centralizations.

Finally, we did not find evidence that network modularity was correlated between the two sampling methods. Network modularity reflects the degree to which animals form clusters of social interactions by interacting preferably with partners belonging to their own clusters compared to partners from other clusters. For this reason, this network metric is commonly assessed in prosocial behaviours such as grooming and huddling [42], whereby behaviours tend to be directed to preferred partners based on long-term affiliations dictated by, for instance, the degree of social bonds [6], or kinship [43]. Aggressive interactions, in contrast, tend to be less modular/clustered as they tend to be distributed more dynamically and may be affected by multiple factors, such as food distribution, or seasonality.

In conclusion, our analysis suggests the use of ABS as a suitable alternative to FAS, particularly if researchers are interested in local network measures, such as degree, eigenvector or betweenness as this seems the most cost-effective method: it allows researchers to collect data on multiple dyads in a shorter amount of time, compared to FAS, while providing similar network metrics as FAS. ABS is likely to be a particularly suitable sampling method for infrequent behaviours such as huddling interactions in despotic species. Finally, we found limited evidence that the degree of despotism/tolerance of a species affects the reliability of the sampling method used to construct social networks. Overall, our results may provide researchers with new guidance on whether to use FAS or ABS to collect their social network data.

## Data Availability

https://figshare.com/projects/Effect_of_behavioural_sampling_methods_on_local_and_global_social_network_metrics_A_case-study_of_three_macaque_species/166205. Supplementary material is available online [44].
